# Gastrodin overcomes chemoresistance via inhibiting Skp2-mediated glycolysis

**DOI:** 10.1038/s41420-023-01648-y

**Published:** 2023-10-02

**Authors:** Li Xie, Jinzhuang Liao, Wenbin Liu, Ruirui Wang, Xiaoying Li, Wei Li, Zhongsu Zhou

**Affiliations:** 1 Department of Head and Neck Surgery, Hunan Cancer Hospital/The Affiliated Cancer Hospital of Xiangya School of Medicine, Central South University, Changsha, Hunan 410013 China; 2 Department of Radiology, The Third Xiangya Hospital, Central South University, Changsha, Hunan 410013 China; 3 Department of Pathology, Hunan Cancer Hospital, Changsha, Hunan 410013 China; 4 The Third Hospital of Changsha, Changsha, Hunan 410015 China

**Keywords:** Oral cancer, Drug development

## Abstract

Aerobic glycolysis, a typical phenotype in human tumors, is associated with tumor progression and chemotherapy resistance. The present study demonstrated that cisplatin-resistant oral squamous cell carcinoma (OSCC) cells exerted a stronger glycolysis ability, which was associated with hexokinase 2 (HK2) overexpression. Additionally, the tumor growth of OSCC cells was delayed in vivo and the glycolysis was notably decreased following HK2 knockdown. The natural compound screening revealed that gastrodin could be an effective candidate for OSCC therapy since it inhibited HK2-mediated glucose metabolism and promoted endogenous OSCC cell apoptosis. Furthermore, gastrodin could bind to protein kinase B (Akt) and suppress its activity, thus downregulating HK2 at the transcriptional level. Additionally, S-phase kinase-associated protein 2 (Skp2) was highly expressed in OSCC cells, while K63-linked ubiquitination of Akt was inhibited in Skp2-depleted cisplatin-resistant OSCC cells. Gastrodin could also inhibit the cisplatin resistance of OSCC cells in vivo, particularly when combined with the Skp2 inhibitor, SZL P1-41. Overall, the aforementioned finding suggested that targeting the Skp2-Akt axis could be a potential therapeutic strategy for treating OSCC and overcoming chemoresistance.

## Facts

- Oral squamous cell carcinoma (OSCC) is characterized by high morbidity and mortality, which is a severe threat to the health of humans.
- Cisplatin-based chemotherapy resistance is responsible for treatment failure in advanced OSCC patients.
- Promoting sensitization to chemotherapy is an important measure to improve the efficacy of chemotherapy.
- Gastrodin is a potential antitumor agent in glioblastoma cells. However, the efficacy of Gastrodin on OSCC is unclear.

## Open questions

- What is the mechanism of acquired cisplatin chemoresistance of OSCC?
- Can we find the chemical candidate that promotes sensitivity to chemotherapy?

## Introduction

Oral squamous cell carcinoma (OSCC), characterized by high morbidity and mortality rates, is a prevalent type of cancer worldwide. OSCC is associated with several risk factors, including betel nut chewing, human papillomavirus infection, alcohol, and tobacco consumption [1–3]. The most common OSCC treatment approaches include surgery, chemotherapy, radiotherapy, targeted therapy and immunotherapy [4]. Despite continuous improvements in treatment, patients with OSCC still suffer from poor prognosis mainly due to late diagnosis and lack of effective treatment strategies [5]. Several chemotherapeutic drugs, such as docetaxel and cisplatin, are used for advanced OSCC treatment. However, acquired drug resistance is one of the major obstacles contributing to treatment failure [6, 7]. Therefore, uncovering the resistance mechanism, identifying new targets, and developing novel drugs is in urgent demand for OSCC treatment.

Cancer cell metabolism is characterized by aerobic glycolysis [8]. The first step in the glycolytic process is catalyzed by the most influential rate-limiting enzyme, namely hexokinase (HK), which irreversibly promotes the phosphorylation of glucose to glucose-6-phosphate [9]. It has been reported that the HK2 isozyme is frequently upregulated in multiple tumors, including hepatocellular carcinoma, glioblastoma, prostate, breast and lung cancer [10, 11]. Previous studies demonstrated that HK2 overexpression could promote the growth and immortalization of tumor cells via regulating glucose metabolism [11]. Additionally, HK2 expression was closely associated with the pathological stage, metastasis, acquired drug resistance and poor prognosis [12, 13]. Furthermore, accumulating evidence has suggested that targeted inhibition of HK2 or HK2 downregulation can inhibit tumor growth and restore the sensitivity of tumor cells to therapy [14, 15]. Therefore, targeting HK2 could still be an effective strategy for treating different types of human tumors, including OSCC.

Accumulating studies on plant extracts have suggested that natural compounds are well tolerated, with excellent biological activities and low toxicity [16]. The main bioactive component of Gastrodia elata, gastrodin, is a traditional Chinese medicinal herb commonly used to treat central nervous system diseases [17]. Gastrodin exhibits several pharmacological activities, including anti-inflammatory, antipsychotic, anti-fibrotic, antioxidant, antiepileptic, anticonvulsant, antitumor effects [18]. A previous study manifested that gastrodin could activate the AMP-activated protein kinase pathway to improve non-alcoholic fatty liver disease [19]. Furthermore, gastrodin could attenuate inflammatory responses and the migration of activated microglia via regulating the neurogenic locus notch homolog protein 1 related pathways [20]. In addition, another study revealed that gastrodin, as an auxiliary agent, could enhance the immunogenicity of melanoma vaccines [21]. Additionally, gastrodin could increase the cytotoxic effects of drugs in glioblastoma cells via inducing oxidative stress-related apoptosis [22]. However, little is known about the anti-tumor effects of gastrodin, and its antitumor efficacy on OSCC remains elusive. In the present study, the anti-tumor activity of gastrodin and its underlying mechanism in OSCC were investigated.

The current study aimed to clarify whether gastrodin could be a promising antitumor agent for treating OSCC. Therefore, the effect of gastrodin on OSCC cells and its underlying mechanism of action were explored both in vitro and in vivo.

## Results

### HK2 is upregulated in cisplatin-resistant OSCC cells

**Fig. 1:**
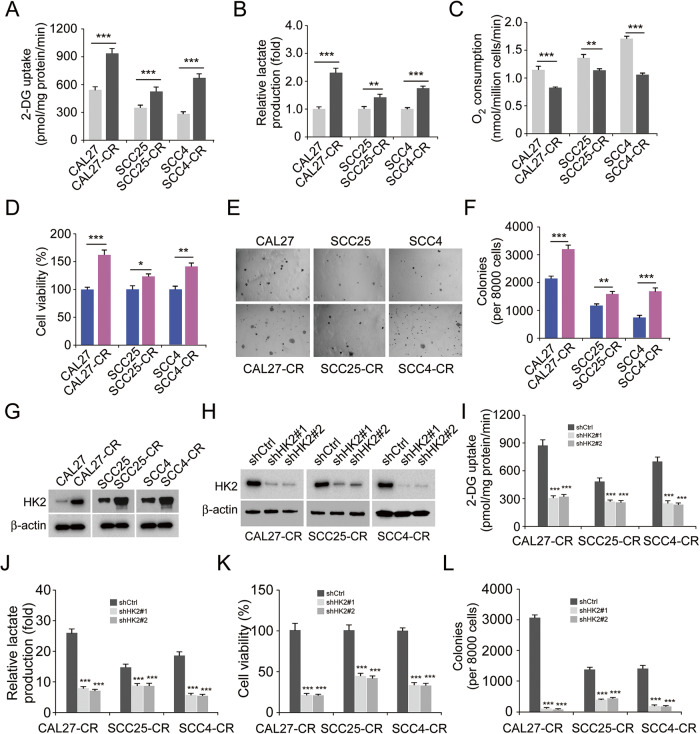
Cisplatin-resistant oral squamous cell carcinoma (OSCC) cell overexpressed HK2. 2-DG uptake (**A**), lactate production (**B**), and O_2_ consumption (**C**) was standardized in three pairs of OSCC cell lines CAL27/CAL27-CR, SCC25/SCC25-CR and SCC4/SCC4-CR subjected to normoxic conditions. **p < 0.01, ***p < 0.001. The cell viability (**D**) and colony formation (**E**) of CAL27/CAL27-CR, SCC25/SCC25-CR and SCC4/SCC4-CR cells were detected using MTS and anchorage-independent analysis, respectively. *p < 0.05, **p < 0.01, ***p < 0.001. **F** The quantitative number of colonies of CAL27/CAL27-CR, SCC25/SCC25-CR, and SCC4/SCC4-CR cells in soft agar. **p < 0.01, ***p < 0.001. **G** HK2 expression was measured using immunoblotting (IB) assay in chemoresistant OSCC cells and parallel parental cells. **H** HK2-depleted CAL27-CR, SCC25-CR and SCC4-CR cells were subjected to IB assay. Normalized 2-DG uptake (**I**) and lactate production (**J**) in HK2-depleted CAL27-CR, SCC25-CR, and SCC4-CR cells subjected to normoxic conditions. ***p < 0.001. **K** The cell viability of CAL27-CR, SCC25-CR and SCC4-CR cells was measured using MTS analysis. ***p < 0.001. **L** The quantitative number of colonies of HK2-depleted CAL27-CR, SCC25-CR, and SCC4-CR cells in soft agar. ***p < 0.001.

Emerging evidence has suggested that cisplatin resistance is a critical factor for chemotherapy failure in OSCC [23]. To further investigate this phenotype, the cisplatin-resistant OSCC cells, CAL27-CR, SCC25-CR, and SCC4-CR, were established from parallel parental cells CAL27, SCC25, and SCC4, respectively. The efficacy of glycolysis in the aforementioned three pairs of OSCC cells was detected under normoxic conditions. The data suggested that chemoresistant OSCC cells possessed a higher glycolysis capacity, since 2-deoxy-d-glucose (2-DG) uptake and lactate production were enhanced in these cells compared with parental cells (Fig. 1A, B). Moreover, the oxygen consumption rate was reduced in chemoresistant OSCC cells, indicating that oxidative phosphorylation in cisplatin-resistant cells was suppressed (Fig. 1C). The MTS results indicated that the cell viability of chemoresistant OSCC cells was higher compared with parental cells (Fig. 1D). The colony formation ability of chemoresistant OSCC cells was also apparently higher than parental cells (Fig. 1E, F). HK2 is one of the rate-limiting enzymes of glycolysis. It was hypothesized that HK2 could play a significant role in chemoresistant OSCC cells. Therefore, the protein expression levels of HK2 were detected in the CAL27 and cisplatin resistant CAL27-CR, SCC25 and cisplatin resistant SCC25-CR and SCC4 and cisplatin resistant SCC4-CR cells. The IB data suggested that HK2 was upregulated in the chemoresistant cell lines CAL27-CR, SCC25-CR, and SCC4-CR (Fig. 1G). Moreover, CAL27 and CAL27-CR cells expressed a higher protein level of HK2 (Fig. S1). The aforementioned findings indicated that the chemoresistant cells exhibited a stronger glycolysis potential compared with the parental cells. To ascertain whether cisplatin resistance was mediated by HK2 upregulation, chemoresistant HK2-depleted OSCC cells were established (Fig. 1H). Subsequently, the efficacy of glycolysis was assessed in chemoresistant HK2-depleted OSCC cells under normoxic conditions. The data suggested that 2-DG uptake and lactate production were decreased after HK2 knockdown (Fig. 1I, J). In addition, the cell viability and colony formation ability of chemoresistant HK2-depleted OSCC cells were significantly attenuated (Fig. 1K, L). Overall, the above findings indicated that HK2 was upregulated in cisplatin-resistant OSCC cells, while HK2 knockdown could suppress OSCC cells.

### HK2 regulates the tumorigenic capability of cisplatin-resistant OSCC cells

**Fig. 2:**
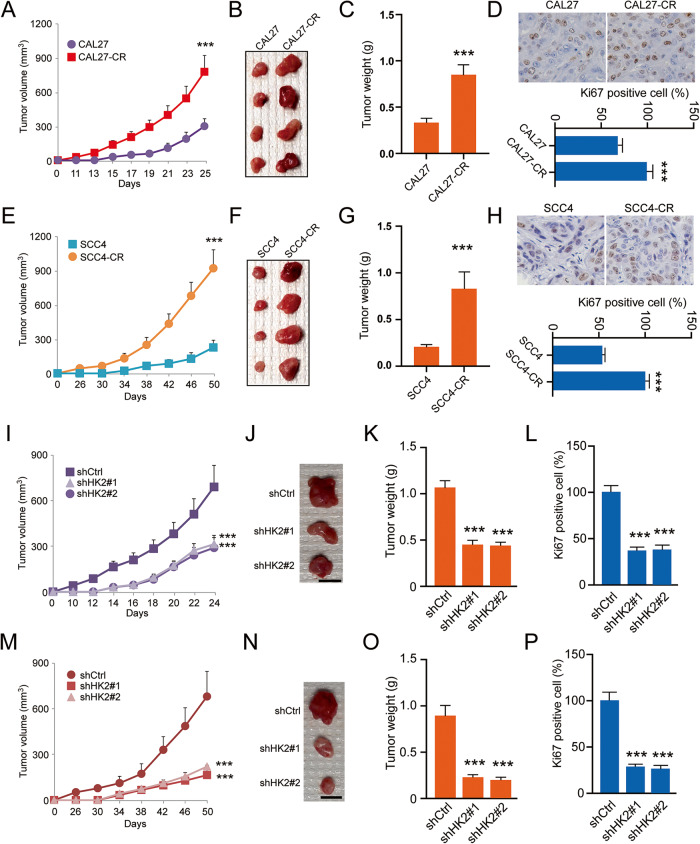
HK2 expression is an essential factor for the tumorigenic capabilities of OSCC cells to chemoresistance. The tumorigenic capabilities of CAL27 and CAL27-CR cells in vivo. **A** tumor volume; **B** tumor mass; **C** tumor weight. ***p < 0.001. **D** The Ki-67 protein levels were assayed using IHC staining. Below, qualification. ***p < 0.001. The tumorigenic capabilities of SCC4 and SCC4-CR cells in vivo. **E** tumor volume; **F** tumor mass; **G** tumor weight. ***p < 0.001. **H** The Ki-67 protein levels were assayed using IHC staining. Below, qualification. ***p < 0.001. For (**D**) and (**H**), scale bar, 25 μm. The tumorigenic capabilities of CAL27-CR cells expressing of shCtrl or shHK2 in vivo. **I** tumor volume; **J** tumor mass; **K** tumor weight. ***p < 0.001. **L** The qualification assessment of IHC staining about Ki-67 in CAL27-CR-derived tumors with shCtrl or shHK2. ***p < 0.001. The tumorigenic capabilities of SCC4-CR cells expressing of shCtrl or shHK2 in vivo. **M** tumor volume; **N** tumor mass; **O** tumor weight. ***p < 0.001. For (**B**), (**F**), (**J**), and (**N**) scale bar, 1 cm. **P** The qualification assessment of IHC staining about Ki-67 in HK2-silenced SCC4-CR-derived tumors. ***p < 0.001.

To determine whether HK2 affects tumorigenesis of cisplatin-resistant OSCC in vivo, xenograft mouse models were established using CAL27/CAL27-CR and SCC4/SCC4-CR cells. The results demonstrated that tumor volume (Fig. 2A), mass (Fig. 2B), and weight (Fig. 2C) were increased more rapidly in CAL27-CR-derived tumors than CAL27-derived tumors. In addition, IHC data revealed that the protein expression levels of Ki-67 and the number of Ki-67 positive cells were notably higher in CAL27-CR-derived tumors (Fig. 2D). Consistently, SCC4-CR-derived tumors displayed similar development efficacy in vivo (Fig. 2E–G). The IHC results were consistent with those observed in CAL27-CR-derived tumors (Fig. 2H). Subsequently, another xenograft mouse model was established using HK2-silenced CAL27-CR and SCC4-CR cells. The results showed that the in vivo growth of CAL27-CR-derived xenograft tumors was markedly delayed following HK2 silencing (Fig. 2I–K). The IHC data revealed that the number of Ki-67 positive cells was significantly decreased in HK2-silenced CAL27-CR-derived tumors (Fig. 2L). Consistently, the same growth rate (Fig. 2M–O) and IHC results (Fig. 2P) were obtained in HK2-silenced SCC4-CR-derived tumors. These findings indicated that HK2 is required for the tumorigenic capability of cisplatin-resistant OSCC cells.

### Gastrodin downregulates HK2 to suppress glycolysis in cisplatin-resistant OSCC cells

**Fig. 3:**
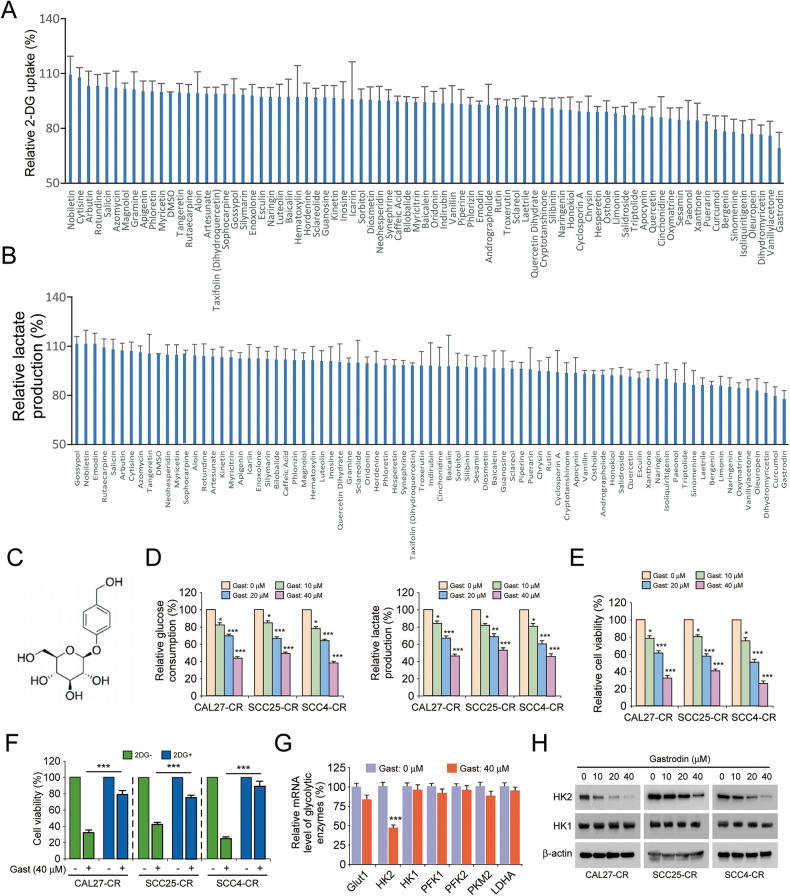
Gastrodin suppresses glycolysis of cisplatin-resistant OSCC cells. 2-DG uptake (**A**) and lactate production (**B**) of SCC25-CR cells was measured using glycolysis assay after treatment with 78 screened compounds (20 µM). **C** Chemical structure of gastrodin. **D** Glucose consumption (left) and lactate production (right) of cisplatin-resistant OSCC cell lines CAL27-CR, SCC25-CR and SCC4-CR were detected after treatment with DMSO control or various concentrations of gastrodin. *p < 0.05, **p < 0.01, ***p < 0.001. **E** The cell viability of CAL27-CR, SCC25-CR and SCC4-CR cells was tested by MTS assay following treatment with DMSO control or various concentrations gastrodin for 24 h. *p < 0.05, ***p < 0.001. **F** The cell viability of CAL27-CR, SCC25-CR, and SCC4-CR cells was tested by MTS assay following treatment with 2-DG, gastrodin, or both in combination. ***p < 0.001. **G** The mRNA level of glycolytic enzymes was measured using RT-qPCR assay after gastrodin (40 µM) treatment. ***p < 0.001. **H** The expression of HK2 and HK1 of CAL27-CR, SCC25-CR, and SCC4-CR cells were measured using IB assay after treatment with DMSO control or various concentrations gastrodin.

To identify whether there are natural compounds that could inhibit glycolysis and HK2 expression of cisplatin-resistant OSCC cells. The customized natural compound pool was next screened. We found that gastrodin could reduce 2-DG uptake and lactate production in SCC25-CR cells by ~25% (Fig. 3A, B). Therefore, gastrodin (Fig. 3C) was selected for the follow-up experiments. To determine whether gastrodin could suppress glycolysis, the cisplatin-resistant cells CAL27-CR, SCC25-CR, and SCC4-CR were treated with different concentrations of gastrodin. The data suggested that gastrodin dose-dependently decreased glucose consumption and lactate production (Fig. 3D). Gastrodin could also inhibit the viability of CAL27-CR, SCC25-CR, and SCC4-CR cells in a dose-dependent manner (Fig. 3E). Subsequently, CAL27-CR, SCC25-CR, and SCC4-CR cells were treated with DMSO (control group), gastrodin, 2-DG, or combination of gastrodin and 2-DG. The data revealed that gastrodin could attenuate the cell viability of cisplatin-resistant OSCC cells (Fig. 3F). Additionally, the inhibitory effect of gastrodin could be diminished following treatment with 2-DG (Fig. 3F). Subsequently, the mRNA expression levels of glycolytic enzymes were detected in gastrodin-treated cisplatin-resistant OSCC cells. The results indicated that the mRNA expression levels of HK2 in cisplatin-resistant OSCC cells were significantly decreased following treatment with gastrodin (Fig. 3G). Furthermore, IB data showed that the protein expression levels of HK2 in cisplatin-resistant OSCC cells were diminished by gastrodin in a dose-dependent manner (Fig. 3H). These findings suggested that gastrodin could downregulate HK2 to suppress glycolysis in cisplatin-resistant OSCC cells.

### Gastrodin activates endogenous apoptosis signaling in cisplatin-resistant OSCC cells

**Fig. 4:**
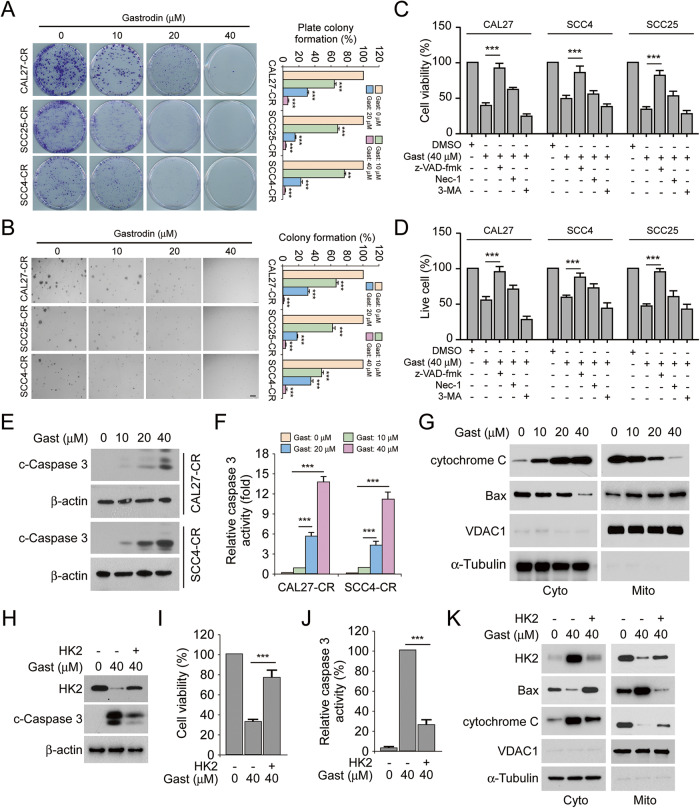
Gastrodin induces endogenous apoptosis and decreases the protein level of HK2. **A** Colony formation of CAL27-CR, SCC25-CR, and SCC4-CR cells treated with various concentrations gastrodin were measured by plate colony formation assay. **p < 0.01, ***p < 0.001. **B** Anchorage-independent growth of CAL27-CR, SCC25-CR, and SCC4-CR cells treated with various concentrations gastrodin were detected using soft agar assay. ***p < 0.001. **C** The viability of OSCC cells following various inhibitors and gastrodin (40 µM) treatment was evaluated using MTS analysis. ***p < 0.001. **D** The number of live cells in various inhibitors- and gastrodin-treated (40 µM) OSCC cells were measured using trypan blue exclusion analysis. ***p < 0.001. The protein levels of cleaved-caspase 3 (**E**) and caspase 3 activity (**F**) in CAL27-CR and SCC4-CR cells after treatment with various doses gastrodin. ***p < 0.001. **G** Isolating subcellular fractions of CAL27-CR cells treated with various doses gastrodin for IB assay. HK2 was transfected into CAL27-CR cells and the cells were treated with gastrodin (40 µM) for 24 h, followed by IB analysis (**H**), MTS analysis (**I**) and Caspase 3 activity assay (**J**). ***p < 0.001. **K** CAL27-CR cells were treated with gastrodin for 24 h following transfecting HK2. Separating subcellular fractions for IB assay.

The intracellular localization of HK2 is closely associated with cell apoptosis and survival. Colony formation assays showed that gastrodin dose-dependently suppressed the plate and soft agar colony formation ability of cisplatin-resistant OSCC cells (Fig. 4A, B). More specifically, treatment of OSCC cells with 40 μM gastrodin decreased colony formation by >90% (Fig. 4A, B). OSCC cells were then pretreated with the cell death inhibitors, z-VAD-fmk, Nec-1 or 3-MA. The data suggested that z-VAD-fmk could restore the viability of gastrodin-treated OSCC cells. However, Nec-1 and 3-MA did not affect OSCC cell viability (Fig. 4C). Consistently, the number of live cells was notably increased following pretreatment with z-VAD-fmk, but not with Nec-1 or 3-MA (Fig. 4D). Subsequently, IB and caspase 3 activity assays were performed. Data manifested that the protein expression levels of cleaved-caspase 3 and caspase 3 activity were enhanced with increasing doses of gastrodin in CAL27-CR and SCC4-CR cells (Fig. 4E, F). Additionally, treatment of CAL27-CR cells with increasing gastrodin doses promoted the translocation of cytochrome C from mitochondria to the cytoplasm (Fig. 4G). Furthermore, Bax protein levels were upregulated in the mitochondria of OSCC cells treated with gastrodin (Fig. 4G). These results indicated that gastrodin could induce endogenous apoptosis. Combined with previous findings, it was hypothesized that HK2 could be necessary for gastrodin-induced cell apoptosis. HK2 was next overexpressed in CAL27-CR cells. The IB data suggested that HK2 overexpression reduced the protein expression levels of cleaved-caspase 3 (Fig. 4H). In addition, HK2 overexpression restored cell viability and diminished caspase 3 activity in gastrodin-treated CAL27-CR cells (Fig. 4I, J). Additionally, the IB data verified that the gastrodin-induced intrinsic apoptosis was inhibited by HK2 overexpression (Fig. 4K). Overall, the above results suggested that gastrodin was depended on HK2 to activate endogenous apoptosis.

### Gastrodin binds to Akt to suppress Akt activation

**Fig. 5:**
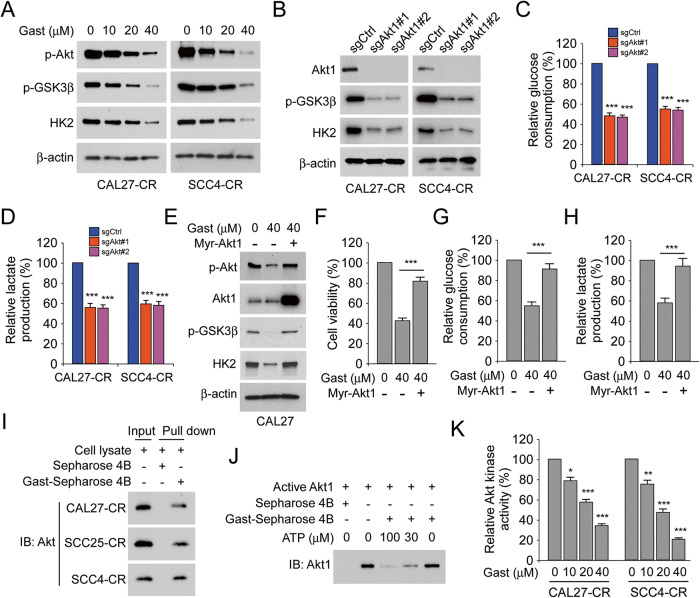
Gastrodin suppresses Akt activation and directly binds to Akt. **A** The expression of p-Akt, p-GSK3β, and HK2 in CAL27-CR and SCC4-CR cells were measured using IB assay following gastrodin treatment. **B** The expression of Akt1, p-GSK3β, and HK2 in CAL27-CR and SCC4-CR cells expressing sgCtrl and sgAkt was detected by IB assay. The cell culture medium of CAL27-CR and SCC4-CR cells with the sgCtrl, and sgAkt steady expression was used for glucose consumption (**C**) and lactate production (**D**) assay. ***p < 0.001. **E**–**H** CAL27 cells were transfected Myr-Akt1 for 24 h and then were treated with gastrodin for 24 h. **E** Whole-cell lysates were subjected to IB analysis to detect the protein levels of p-Akt, Akt1, p-GSK3β, and HK2. **F** The cell viability was evaluated by MTS assay. ***p < 0.001. The cell culture medium of the cells was used for glucose consumption (**G**) and lactate production (**H**) assay. ***p < 0.001. **I** Lysates from CAL27-CR, SCC25-CR and SCC4-CR cells and Sepharose 4B or Gast-Sepharose 4B beads were co-incubated to perform pull-down assay. The complexes were used for IB assay. **J** Active Akt1 was incubated with various doses of ATP (30 or 100 μM) overnight, followed by incubation with Gast-Sepharose 4B beads for 4 h. The protein levels of Akt1 were measured using IB assay after washing and boiling the beads with loading buffer. **K** Akt kinase activity was detected by Akt Kinase Activity Assay Kit in CAL27-CR and SCC4-CR cells with various doses of gastrodin treatment. *p < 0.05, **p < 0.01, ***p < 0.001.

Akt is one of the key upstream regulators of HK2 [24, 25]. Therefore, the expression levels of p-Akt and its downstream proteins were determined. The results showed that p-Akt, p-GSK3β and HK2 were dose-dependently downregulated in gastrodin-treated CAL27-CR and SCC4-CR cells (Fig. 5A). Furthermore, the phosphorylation of GSK3β and HK2 expression was reduced in Akt-depleted CAL27-CR and SCC4-CR cells (Fig. 5B). Additionally, glucose consumption and lactate production were also decreased in Akt-depleted CAL27-CR and SCC4-CR cells (Fig. 5C, D). Subsequently, CAL27 cells were transfected with Myr-Akt1 to verify whether Akt overexpression could reverse the aforementioned phenotype. The results indicated that persistent ectopic Akt overexpression could restore the phosphorylation levels of Akt and GSK3β, and HK2 expression in gastrodin-treated CAL27 cells (Fig. 5E). The MTS and glycolysis assay results also demonstrated that Akt overexpression in gastrodin-treated CAL27 cells restored cell viability and increased glucose consumption and lactate production (Fig. 5F–H). Additionally, pull-down assays were carried out to evaluate the interaction between gastrodin and Akt. The data indicated that Akt could interact with the Gast-Sepharose 4B beads complex and not with Sepharose 4B beads only (Fig. 5I). Furthermore, ATP competition assay results showed that the interaction between gastrodin and Akt was reduced in the presence of ATP, indicating that gastrodin might disturb the interaction between Akt and ATP (Fig. 5J). Additionally, the Akt kinase activity assay results showed that the activity of Akt was gradually attenuated in CAL27-CR and SCC4-CR cells treated with increasing gastrodin doses (Fig. 5K). Overall, the aforementioned findings suggested that gastrodin, an ATP competitive inhibitor, could directly bind to Akt and inhibit its activity.

### Skp2 mediated K63-linked ubiquitination of Akt and combination of gastrodin and SZL P1-41 can inhibit glycolysis in cisplatin-resistant OSCC cells

**Fig. 6:**
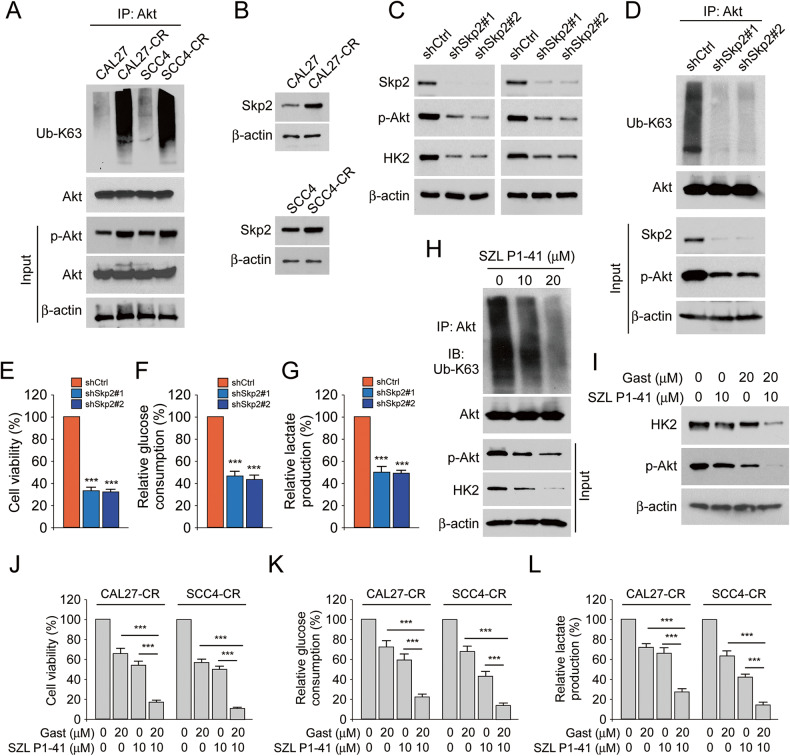
Skp2 regulated polyubiquitination of Akt and glycolysis which could be inhibited by gastrodin. **A** IB assay for IP-mediated Akt polyubiquitination in CAL27/CAL27-CR and SCC4/SCC4-CR cells. **B** IB analysis of the expression of Skp2 in CAL27/CAL27-CR and SCC4/SCC4-CR cells. **C** IB analysis of the expression of Skp2, p-Akt and HK2 in Skp2-silenced CAL27-CR and SCC4-CR cells. **D** IB assay for IP-mediated Akt polyubiquitination in CAL27-CR cells with Skp2 knockdown or control. **E** The cell viability of CAL27-CR cells with Skp2 knockdown or control was evaluated using MTS analysis. ***p < 0.001. The cell culture medium of CAL27-CR cells with Skp2 knockdown or control was used for glucose consumption (**F**) and lactate production (**G**) assay. ***p < 0.001. **H** IB assay for IP-mediated Akt polyubiquitination in SZL P1-41-treated CAL27-CR cells. **I–L** CAL27-CR and SCC4-CR cells were treated with DMSO, SZL P1–41 (10 µM), gastrodin (20 µM) or both in combination. **I** IB analysis of p-Akt and HK2 expression. **J** The cell viability was evaluated using MTS analysis. ***p < 0.001. The cell culture medium of the cells was used for glucose consumption (**K**) and lactate production (**L**) assay. ***p < 0.001.

Akt ubiquitination plays a crucial role in Akt activation [26]. The results manifested that the polyubiquitination level of Akt Ub-K63 was enhanced in CAL27-CR and SCC4-CR cells compared with the corresponding parental cells (Fig. 6A). In addition, the expression levels of Skp2 were increased in CAL27-CR and SCC4-CR cells (Fig. 6B). Subsequently, CAL27-CR and SCC4-CR cells were stably transfected with shCtrl or shSkp2 and an IB assay was then carried out. The IB data showed that Akt phosphorylation and HK2 expression were decreased in Skp2-silenced cells (Fig. 6C). Additionally, the level of K63-linked polyubiquitination of Akt (Fig. 6D), the cell viability (Fig. 6E), glucose consumption (Fig. 6F) and lactate production (Fig. 6G) were reduced in cisplatin-resistant OSCC cells following Skp2 knockdown. Furthermore, Skp2 inhibitor (SZL P1-41) was used to treat CAL27-CR cells and the results showed that Akt polyubiquitination was dose-dependently attenuated in the cells (Fig. 6H). As shown in Fig. 6I-L, our IB data revealed that co-treatment of cisplatin-resistant OSCC cells with SZL P1-41 and gastrodin notably decreased HK2 expression and inhibited Akt phosphorylation compared with cells treated with each compound alone (Fig. 6I). In addition, cell viability (Fig. 6J), glucose consumption (Fig. 6K) and lactate production (Fig. 6L) were obviously decreased following cell co-treatment with SZL P1-41 and gastrodin compared with SZL P1-41 or gastrodin alone treatment. These findings indicated that overexpressed Skp2 promoted the polyubiquitination level of Akt Ub-K63 and glycolysis in cisplatin-resistant OSCC cells.

### Cell co-treatment with gastrodin and SZL P1-41 exhibits a synergistic inhibitory effect on chemoresistant OSCC cells in vivo

**Fig. 7:**
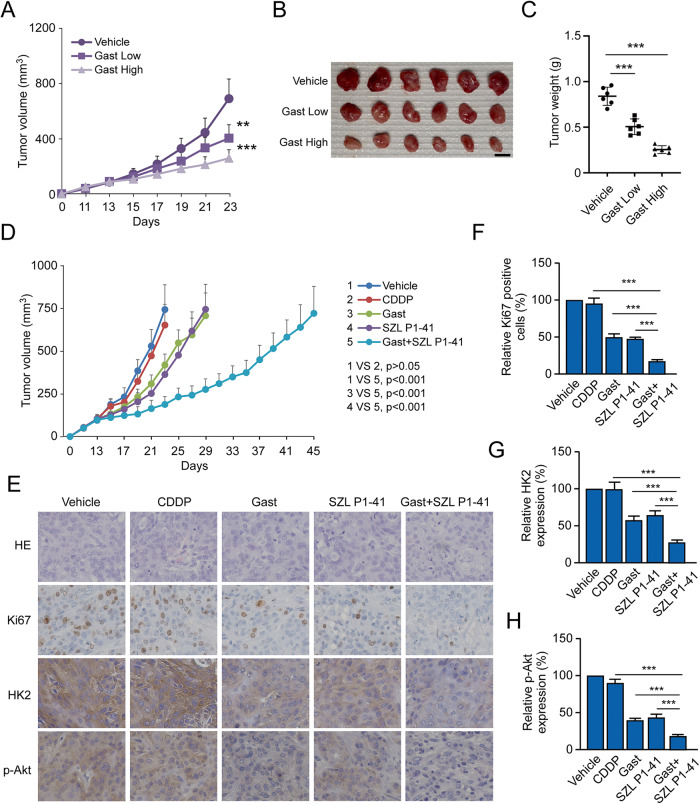
Combination of gastrodin and SZL P1-41 exhibited a better inhibitory effect for the growth of OSCC-derived tumors in vivo. The growth curves (**A**), mass (**B**), and weight (**C**) of tumors derived from CAL27-CR cells after vehicle, gastrodin low and high treatment. **p < 0.01, ***p < 0.001. Scale bar, 1 cm. **D** The growth curves of tumors derived from CAL27-CR cells following vehicle, CDDP, gastrodin, SZL P1-41, or combining gastrodin with SZL P1–41 treatment. **E** Xenograft tumor tissues derived from CAL27-CR cells were used for IHC staining assay of Ki67, HK2, and p-Akt. Scale bar, 25 μm. **F**–**H** Qualification of IHC staining results. ***p < 0.001.

Subsequently, xenograft mouse models derived from CAL27-CR cells were established to verify whether gastrodin could inhibit tumor progression in vivo. As shown in Fig. 7A–C, gastrodin dose-dependently reduced the tumor volume (Fig. 7A), mass (Fig. 7B), and weight (Fig. 7C) of CAL27-CR-derived tumors. Moreover, the hematoxylin-eosin staining of essential organs in gastrodin-treated groups exhibited no apparent abnormalities compared to the vehicle control group (Fig. S2A). Moreover, gastrodin treatment unaffected red blood cells (RBC), WBC, ALT, and AST in mice blood samples (Fig. S2B). These results suggest that gastrodin is well tolerated in vivo. In addition, the in vivo progression of CAL27-CR-derived tumors, after treatment with vehicle, CDDP alone, gastrodin alone, SZL P1–41 alone or combination of gastrodin with SZL P1-41, was investigated. The data suggested that co-treatment with gastrodin and SZL P1-41 significantly delayed tumor growth compared with in vivo treatment with vehicle or other compounds (Fig. 7D). In addition, IHC staining results suggested that Ki67, HK2 and p-Akt were markedly downregulated following co-treatment with gastrodin and SZL P1–41 (Fig. 7E–H). The aforementioned findings indicated that the simultaneous administration of gastrodin and Skp2 inhibitors could significantly suppress tumor growth in vivo.

## Discussion

Oral cancer ranks sixteenth among all malignant tumors worldwide [5]. Among all oral cancer cases, OSCC accounts for ~90%, while its pathogenesis is driven by several factors, such as viral infection, tobacco and alcohol consumption [1, 5]. Accumulating evidence has suggested that glycolysis serves a significant role in the progression of OSCC. For example, E3 ubiquitin-protein ligase NEDD4-like could induce the ubiquitination and degradation of enolase 1 to suppress glucose metabolism in OSCC cells [27]. Another study revealed that the cytoskeleton regulator/heterogeneous ribonuclear protein C/zinc finger E-box-binding homeobox 1 and insulin-like growth factor 2 mRNA-binding protein 2/N6-methyladenosine/HK2 axis could induce abnormal glycolysis in OSCC cells [28, 29]. Period 1 could inhibit glycolysis and cell growth in OSCC [30]. Furthermore, CDK5 regulatory subunit associated protein 2 upregulation was associated with cell metastasis and clinical stage of OSCC [31]. Additionally, circular RNA *PKD2*, an anti-oncogene, could facilitate autophagy related 13-mediated autophagy to improve the sensitivity of OSCC to cisplatin [32]. TNF receptor associated factor 4, an E3 ligase, could stabilize myeloid leukemia 1 (Mcl-1) to promote OSCC radioresistance [33]. The present study demonstrated that HK2 was upregulated in cisplatin-resistant OSCC cells, while the in vitro and in vivo tumorigenic capability of cisplatin-resistant OSCC cells was attenuated following HK2 knockdown. The results also suggested that gastrodin could suppress HK2-mediated glycolysis and tumor progression in OSCC. These findings indicated that aberrant glycolysis was a hallmark of human OSCC.

Compared with normal cells, HK2 was significantly upregulated in cancer cells and notably associated with tumor cell survival and progression [10]. Previous studies also demonstrated that the expression of HK2 was positively regulated by the transcription factors hypoxia-inducible factor-1, BTB domain and CNC homolog, c-Myc and nuclear transcription factor Y, and negatively regulated by KLF transcription factor 14 [11, 34–36]. Additionally, other studies demonstrated that the expression of HK2 was regulated by several microRNAs (miRs), such as miR-185, miR-218, miR-216a-5p, and miR-155, and long intergenic non-protein coding RNA-RoR [10, 36]. Several signaling pathways have also been associated with the regulation of HK2 expression, including the PI3K/Akt [15], Aurora-A/ SRY‑related HMG‑box 18/forkhead box K1 [37], and B7-H3/SATA3 pathways [14]. Currently, the tumorigenicity of HK2 has been associated with two aspects. In the first aspect, the metabolic activity of HK2, as a glucokinase, could play a vital role in immune escape and resistance to radio/chemotherapy (cisplatin, paclitaxel) during tumorigenesis and tumor development [10, 38]. For example, previous studies demonstrated that HectH9 and Skp2, two E3 ligases, could promote the translocation of HK2 to the mitochondria to enhance glycolysis and promote chemoresistance [39, 40]. In the other aspect, HK2 could act as a pure scaffold, which is not associated with its catalytic activity. For instance, a study revealed that HK2 could bind to and inhibit the function of the mechanistic target of rapamycin C1 and promote the activation of uncoordinated 51 like kinase 1 to induce autophagy under glucose starvation conditions [41]. The combination of HK2 with GSK3 and PKA regulatory subunit RIa could promote GSK3 phosphorylation, thus affecting the epithelial mesenchymal transformation and metastasis of tumor cells [42]. Other studies showed that the deletion of HK2 could impair the tumorigenicity of tumor cells, thus restoring their sensitivity to radio/chemotherapy [11, 36]. The results showed that gastrodin could downregulate HK2 and activate endogenous apoptosis signals. HK2 overexpression suppressed gastrodin-induced cell death and restored glycolysis in OSCC cells, based on its catalytic activity. Additionally, Skp2 overexpression also increased the levels of the K63-linked polyubiquitination of Akt in OSCC cells, thus leading to Akt inactivation and HK2 inhibition. In addition, gastrodin could bind to Akt to suppress Akt phosphorylation. Ectopic Akt overexpression reversed the gastrodin-mediated reduction of Akt phosphorylation, HK2 downregulation and glycolysis inhibition in OSCC cells. This finding suggested that gastrodin could be an excellent candidate compound for OSCC therapy.

A previous study showed that Skp2 overexpression was associated with tumor progression and poor prognosis in several tumors, such as ovarian, colorectal, gastric and oral cancer [43]. It has been reported that Skp2 is involved in the regulation of cell cycle, cell apoptosis and senescence, while it promotes tumor progression and radio/chemotherapy resistance [43, 44]. For example, another study demonstrated that the signal transducer and activator of the transcription Skp2/p27 pathway was associated with senescence in glioblastoma cells [45]. Additionally, Mcl-1 was stabilized by Skp2 to promote radioresistance in colorectal cells [44]. Two types of substrates by Skp2 have been identified [43]. The first is the Skp2-mediated K48-linked ubiquitination, which promotes proteolytic substrates such as p21, p27, co-activator-associated arginine methyltransferase 1 and forkhead transcription factor 1 [43, 46, 47]. The other one is the Skp2-mediated K63-linked ubiquitination, which promotes non-proteolytic substrates, such as Akt, yes‑associated protein, ras-related GTP-binding protein A and liver kinase B1 [43]. In addition, previous studies also showed that the expression of Skp2 and its function, such as Skp2-SCF complex formation, proteolysis and mRNA transcription, were regulated by Akt [43]. The current study revealed that Skp2 was upregulated in cisplatin-resistant OSCC cells, while Skp2 silencing attenuated glycolysis and the K63-linked polyubiquitination of Akt, thus inhibiting Akt activation. Additionally, co-treatment with gastrodin and Skp2 inhibitors obviously suppressed the development of tumors derived from cisplatin-resistant OSCC cells in vivo.

In summary, the results of the current study illustrated that the Skp2/Akt/HK2 pathway could promote the resistance of OSCC cells to chemotherapy. Therefore, targeting this pathway could be a potential therapeutic strategy for tumors after chemoresistance. The results also demonstrated that gastrodin could bind to and directly inhibit Akt, thus downregulating HK2 and inhibiting glycolysis in OSCC cells. In addition, gastrodin inhibited the progression of chemoresistance in OSCC cells in vivo, particularly when combined with Skp2 inhibitors (SZL P1-41). Therefore, gastrodin could be a promising tumor sensitizer for OSCC therapy.

## Materials and methods

### Reagents and antibodies

Tris base, NaCl, SDS and DMSO were purchased from MilliporeSigma (St. Louis, MO). Gastrodin, cisplatin (CDDP) and SZL P1-41 were obtained from Selleck Chemicals (Houston, TX). Fetal Bovine Serum (FBS) and cell culture media (DMEM) were purchased from Invitrogen (Grand Island, NY), while Lipofectamine^®^™ 2000 was used for transfection from Thermo Fisher Scientific, Inc. Antibodies against β-actin (#3700), HK2 (#2867), HK1 (#2024), α-tubulin (#2144), Bax (#14796), cleaved-caspase 3 (#9664), cytochrome c (#4280), VDAC1 (#4866), p-GSK3β (#5558), p-Akt (#4060), Akt1 (#2938), Akt (#4685), ubiquitin (#12930), Skp2 (#2652S), cleaved-PARP (#5625), anti-mouse IgG HRP (#7076), and anti-rabbit IgG HRP (#7074) were from Cell Signaling Technology, Inc. Antibodies against Ki67 (ab16667) were products of Abcam.

### Cell culture

293T, SCC25, CAL27, and SCC4 cells were obtained from the American Type Culture Collection. The cisplatin-resistant CAL27-CR, SCC25-CR, and SCC4-CR cell lines were established from the corresponding parental OSCC cells via gradually increasing cisplatin dose and maintaining it for 6 months. Cells were maintained in DMEM supplemented with 10% FBS, and 1% penicillin–streptomycin at 37 °C in a humidified incubator with 5% CO_2_, according to the manufacturer’s instructions, and were routinely screened for mycoplasma infection.

### Glycolysis assay

The glycolysis assay was conducted as previously described [48]. Briefly, OSCC cells seeded into 48-well plates were treated with DMSO (control) or gastrodin for 12 h. The levels of lactate and glucose were determined at the Laboratory of Xiangya Hospital, Changsha, China. The relative lactate production and glucose consumption rates were normalized based on the protein concentration.

### MTS assays

MTS assay was performed as previously described [33]. Briefly, OSCC cells at a density of 3 × 10^3^ cells/well/100 μL were seeded into a 96-well plate, followed by overnight incubation. The cells were then treated with gastrodin for different time points. Cell viability was detected after adding MTS reagent (#G3580, Madison, WI) into the cell culture medium, according to the supplier’s instructions.

### Lentiviral infection

The pLKO.1-shHK2 or pLKO.1-shSkp2 lentiviral plasmids, and PMD2-G and PSPAX2 plasmids were co-transfected into 293T cells to establish HK2- or Skp2-depleted stable cells. Following cell transfection for 72 h, the supernatant containing the viral particles was collected. When reached ~70–80% confluency, OSCC cells were infected with the aforementioned lentiviral particles using polybrene (5 μg/mL). Finally, stable cells were selected following cell treatment with 2 μg/mL puromycin for 2 weeks.

### Establishment of Akt1-depleted stable cell lines

Akt1-depleted stable cells were established as previously described [36]. Briefly, cisplatin-resistant OSCC cells were transfected with sgAkt1 plasmid and cells were then screened following cell treatment with 2 μg/ml puromycin.

### Plate colony formation assay

Cisplatin-resistant OSCC cells were treated with DMSO or gastrodin (10, 20, and 40 μM), followed by incubation in a six-well plate (500 cells/well) under normal conditions for 2 weeks. Visible colonies were fixed with 4% paraformaldehyde and stained with 0.5% crystal violet at 37 °C. Colonies were counted using a light microscope.

### Soft agar assay

Soft agar assay was performed as previously described [49]. Briefly, a total of 8 × 10^3^ OSCC cells or cisplatin-resistant OSCC cells were suspended in 1 mL 0.3% agar with Eagle’s medium (10% FBS) and were then seeded onto the top of the bottom layer of a six-well plate supplemented with 0.6% agar base. Following incubation for two weeks under normal conditions, images of the soft agar colonies were captured and colonies were counted.

### Natural product screening

A library of 78 natural products from Selleck Chemicals was used for screening. Cisplatin-resistant OSCC cells were seeded into a 96-well plate and treated with natural products (20 μM/well) or DMSO (control) for 12 h. The supernatant of the cell culture was then subjected to glycolysis assay, which was performed at the Laboratory of The Xiangya Hospital, Changsha, China. All screened natural products are listed in Fig. 3A, B.

### Trypan blue exclusion assay

Cell number and viability were assessed using a hemocytometer (Neubauer Chambe) and trypan blue staining. Trypan blue staining is used to differentiate dead cells or non-viable (blue) from alive (bright) cells.

### Immunoblotting (IB) assay

IB assay was carried out as previously described [39]. Briefly, a whole-cell extraction (WCE) buffer was formulated using pre-cooled RIPA buffer supplemented with protease inhibitors (#89900; Thermo Fisher Scientific, Inc.). The protein concentration was measured using a BCA protein assay (#23225, Thermo Fisher Scientific, Inc.). Proteins were then supplemented with loading solution with 30 μg WCE and loading buffer, followed by boiling for 5 min at 95 °C. Subsequently, the protein samples were separated by SDS-PAGE and were then electro-transferred onto a PVDF membrane. The membrane was then incubated with 5% non-fat milk in 10% TBST for 50 min at 25 °C. Following incubation with a primary antibody at 4 °C overnight, the membrane was next incubated with the corresponding secondary antibody for 40 min at 25 °C. The protein of interest was visualized using a chemiluminescence solution.

### Ubiquitination analysis

Ubiquitination analysis was conducted as previously described [50]. For immunoprecipitation (IP)-mediated endogenous ubiquitination analysis, cells were lysed using a modified RIPA buffer supplemented with protease inhibitors and N-ethylmaleimide (10 mM). The lysates were sonicated for 30 s, boiled for 15 min at 95 °C, diluted in 0.1% SDS solution supplemented with RIPA buffer, and centrifuged at 4 °C for 15 min at 16,000 *g*. The supernatant was then used for IP analysis. Finally, the ubiquitination of the protein of interest was detected using IB assay.

### Ex vivo pull-down assay

The ex vivo pull-down assay was performed as previously described [49]. Briefly, gastrodin was conjugated with Sepharose 4B beads according to the manufacturer’s instructions. Subsequently, CAL27-CR/SCC25-CR/SCC4-CR cell lysates (400 μg) were incubated with the control or gastrodin-conjugated Gast-Sepharose 4B beads at 4 °C for 12 h. A binding buffer was used to extract beads, which were then subjected to IB assay.

### ATP-competitive binding assay

ATP-competitive binding assays were conducted as previously described [50]. The active Akt and different doses of ATP were pre-incubated at 4 °C overnight. Subsequently, the reaction solution was supplemented with Sepharose 4B or gastrodin-conjugated Sepharose 4B beads and incubated at 4 °C overnight. Beads were then extracted using a wash buffer. IB assay was performed to detect protein binding.

### Akt kinase activity assay

The Akt Kinase Activity Assay kit (ab139436, Abcam) was used to detect total Akt activity in gastrodin-treated OSCC cells according to the manufacturer’s protocols. In brief, lysates from gastrodin-treated CAL27-CR and SCC4-CR cells were homogenized, followed by incubation with ATP and Akt phosphor-specific substrate antibodies in an ELISA plate. Subsequently, each well was supplemented with HRP-conjugated anti-rabbit IgG and TMB solution, followed by treatment with a stop solution. The absorbance was finally measured at a wavelength of 450 nm.

### Xenograft mouse model

All animal experiments were approved by the Institutional Animal Care and Use Committee of the Hunan Cancer Hospital, Changsha, China (approval no. 2021–119). CAL27 (2 × 10^6^ cells), SCC4 (5 × 10^6^ cells), or CAL27-CR (2 × 10^6^ cells) were injected into the right flank of 6-week-old female thymus-free nude mice (*n* = 6). Tumor size and the body weight of mice were recorded every 2 days. When tumor volume reached ~100 mm^3^, the tumor-bearing mice were randomly allocated into three groups (*n* = 6 mice/group). Mice in the control group were injected with vehicle control via intraperitoneal injection (i.p.). Mice in the treatment groups were administrated gastrodin (low gastrodin group, 10 mg/kg every two days, i.p.; high gastrodin group, 30 mg/kg every 2 days, i.p.). For combined treatment, the tumor-bearing mice were randomly divided into the following five groups (*n* = 5 mice/group): i. The vehicle control group (0.5% DMSO/two days, i.p.); ii. the CDDP group (4 mg/kg/4 days, i.p.); iii. the gastrodin group (10 mg/kg/two days, i.p.); iv. the SZL P1-41 group (5 mg/kg/2 days, i.p.); v. the gastrodin (5 mg/kg/two days, i.p.) + SZL P1-41 (3 mg/kg/2 days, i.p.) group. Tumor volume was calculated according to the following formula: Length × width^2^ × 0.5. A fill rate of 30% of the chamber volume per minute with CO_2_ (3 L/min) for 5 min was used to euthanize mice. Euthanasia was further confirmed by cervical dislocation.

### Immunohistochemical (IHC) staining

The tissues derived from xenograft tumors were fixed and IHC assays were carried out as described previously [24]. Briefly, after being deparaffinized and rehydrated, the tissue sections were submerged into sodium citrate buffer (10 mM, pH 6.0) and boiled for 10 min. Subsequently, the tissue sections were incubated with 3% H_2_O_2_ in methanol for 10 min and washed with PBS. The slides were then blocked with 5% goat serum albumin, followed by incubation with a primary antibody for 12 h at 4 °C and then with the corresponding secondary antibody for 45 min at room temperature. Immunoreactivity was visualized using a DAB substrate. Finally, hematoxylin was applied for counterstaining.

### Blood assay

Blood was collected via cardiac puncture of mouse with EDTA coating pipes. The RBC, white blood cells (WBC), alanine aminotransferase (ALT), and aspartate aminotransferase were analyzed in the laboratory of Xiangya Hospital, Central South University.

### Statistical analysis

Data analysis was performed using GraphPad Prism 5 software (GraphPad Software, Inc.) and expressed as the mean ± SD from at least three independent experiments. The differences between or among groups were assessed via Student’s *t* test or ANOVA. *p* < 0.05 was considered to indicate a statistically significant difference.

### Supplementary information

Supplementary material

Original Data File
