# Supplementary material for: Gastrodin overcomes chemoresistance via inhibiting Skp2-mediated glycolysis
Source: Cell Death Discov. 2023 Oct 2;9:364. doi: 10.1038/s41420-023-01648-y (PMC10543462; doi:10.1038/s41420-023-01648-y)
Supplement: Supplementary file 1 — Supplementary material [file 41420_2023_1648_MOESM1_ESM.pdf]

Figure S1

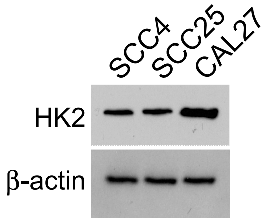

Figure S2

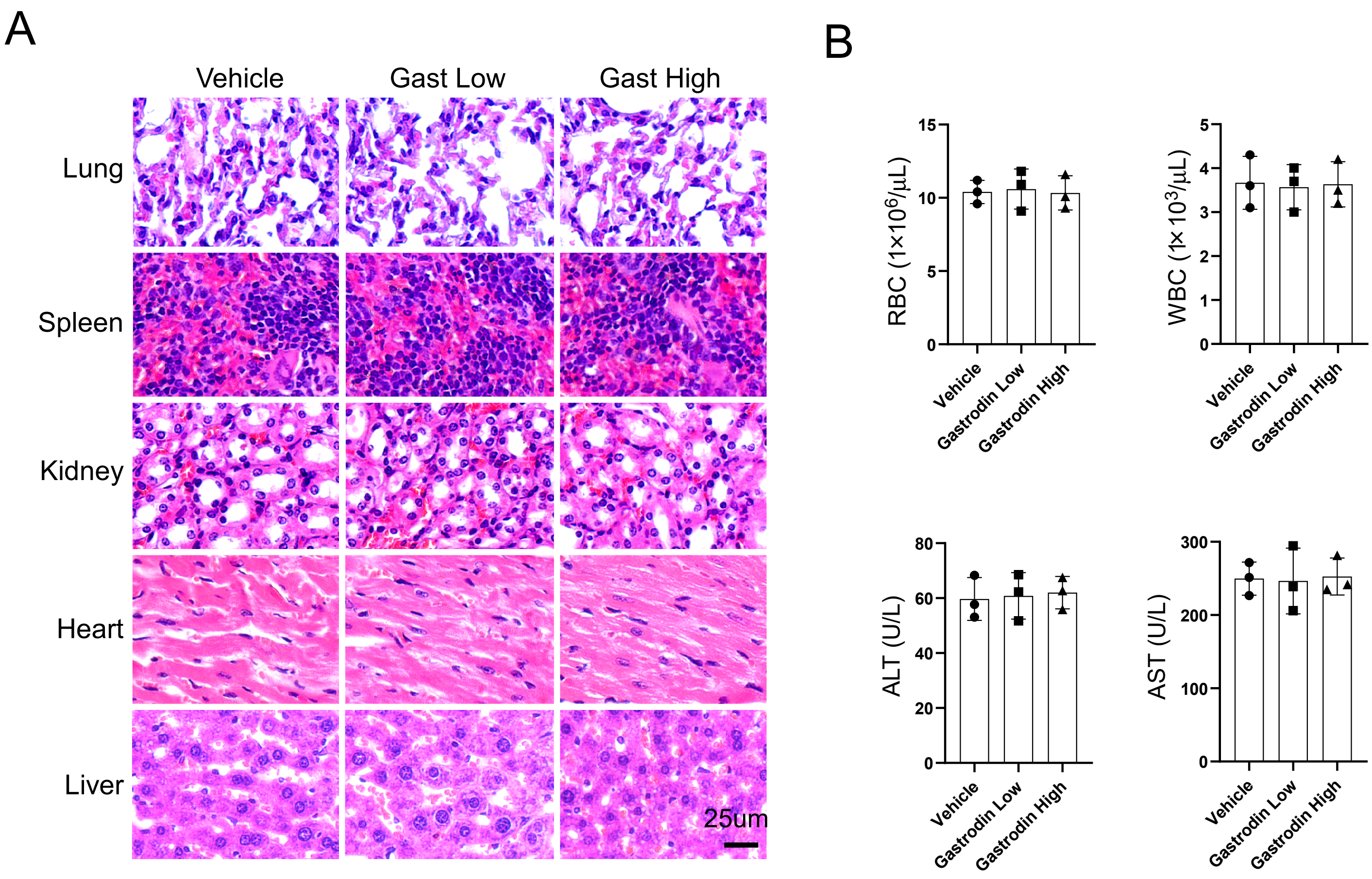

Supplementary Figure 1: SCC4, SCC25, and CAL27 cells were subjected to IB assay to detect HK2 expression.

Supplementary Figure 2: (A) Hematoxylin-eosin staining analysis of organs of CAL27-CR tumor-bearing mice following various doses of gastrodin or vehicle treatment. (B) Mouse blood analysis after various doses of gastrodin or vehicle treatment. RBC, WBC, ALT, and AST levels were analyzed.
