# Supplementary figures and images for: Gastrodin overcomes chemoresistance via inhibiting Skp2-mediated glycolysis
Source: Cell Death Discov. 2023 Oct 2;9:364. doi: 10.1038/s41420-023-01648-y (PMC10543462; doi:10.1038/s41420-023-01648-y)

Figure 1

G

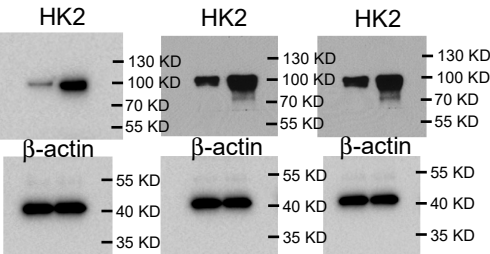

H

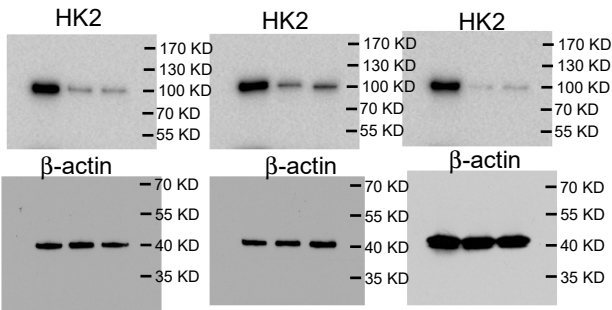

Figure 3

F

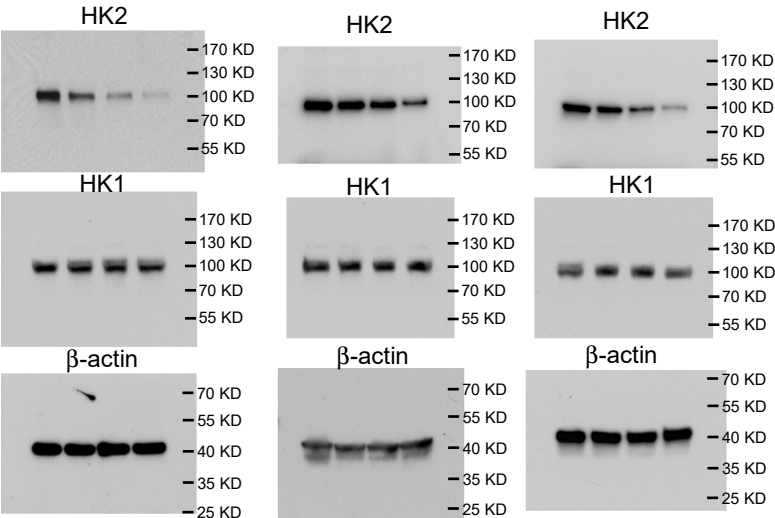

Figure 4

E

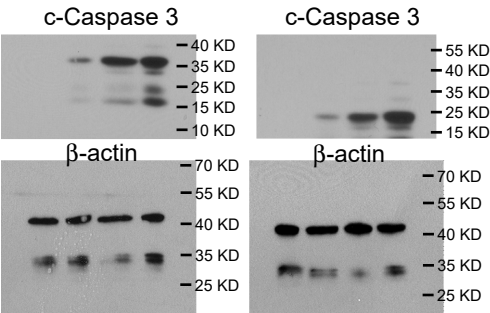

K

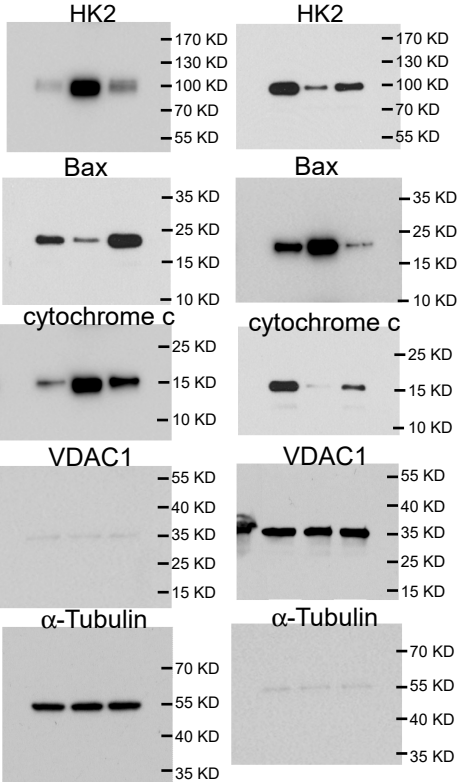

G

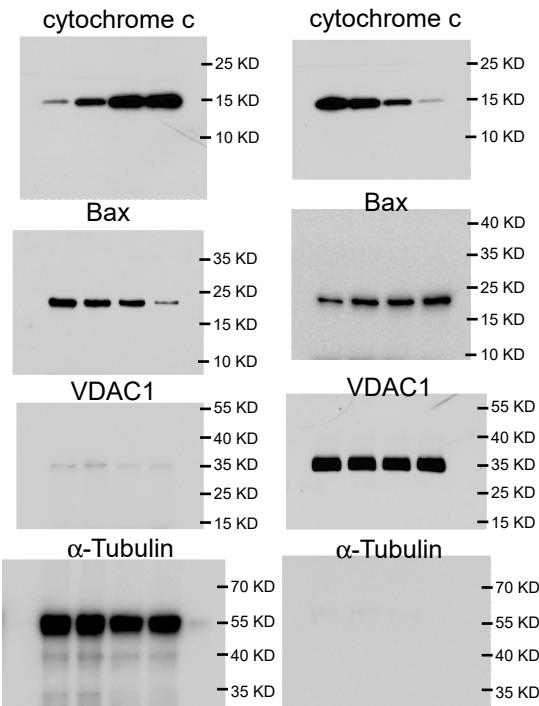

H

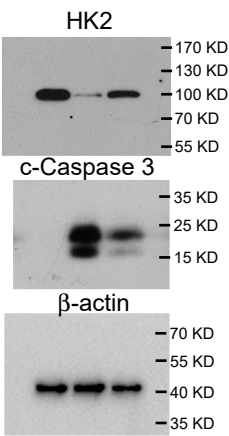

Figure 5

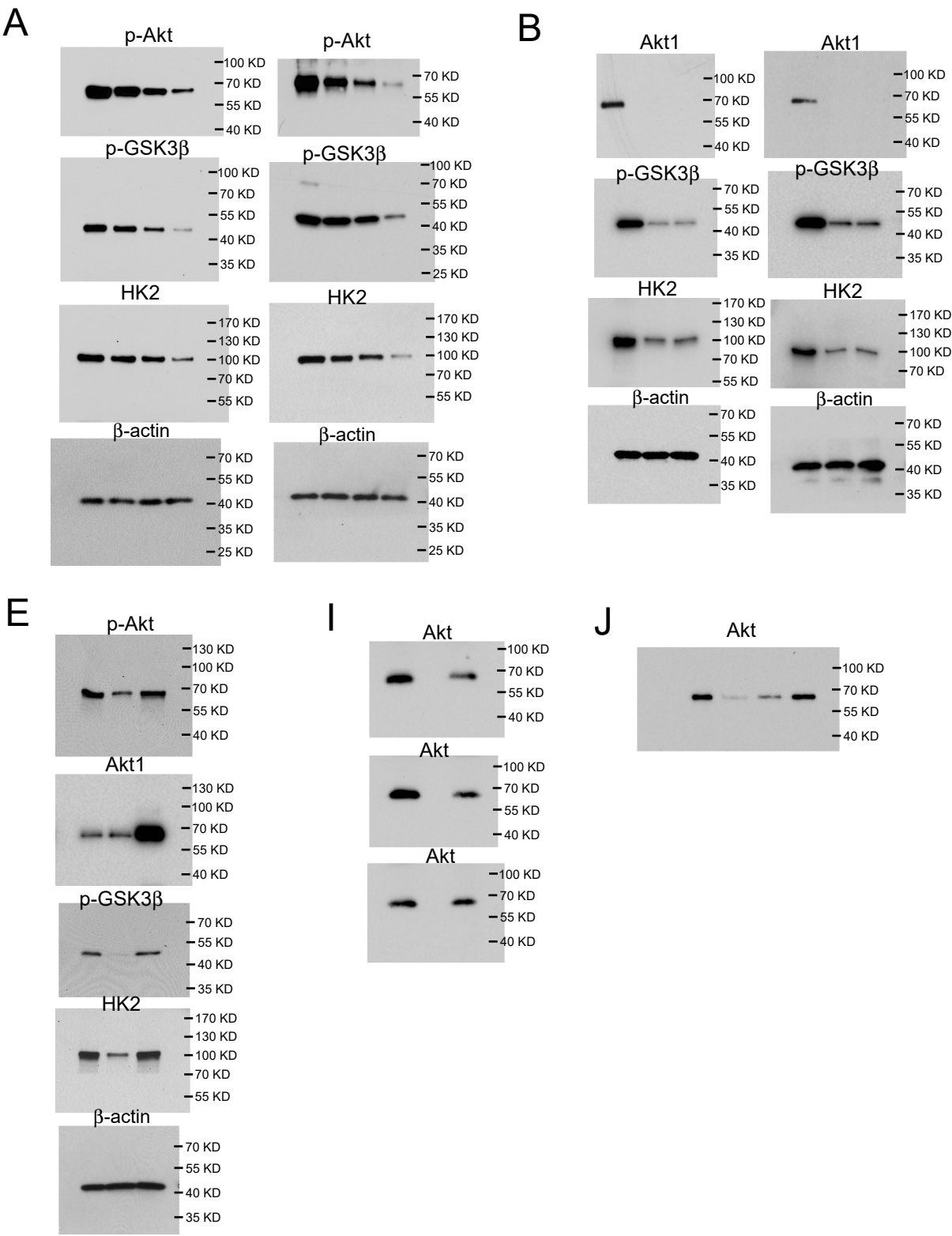

Figure 6

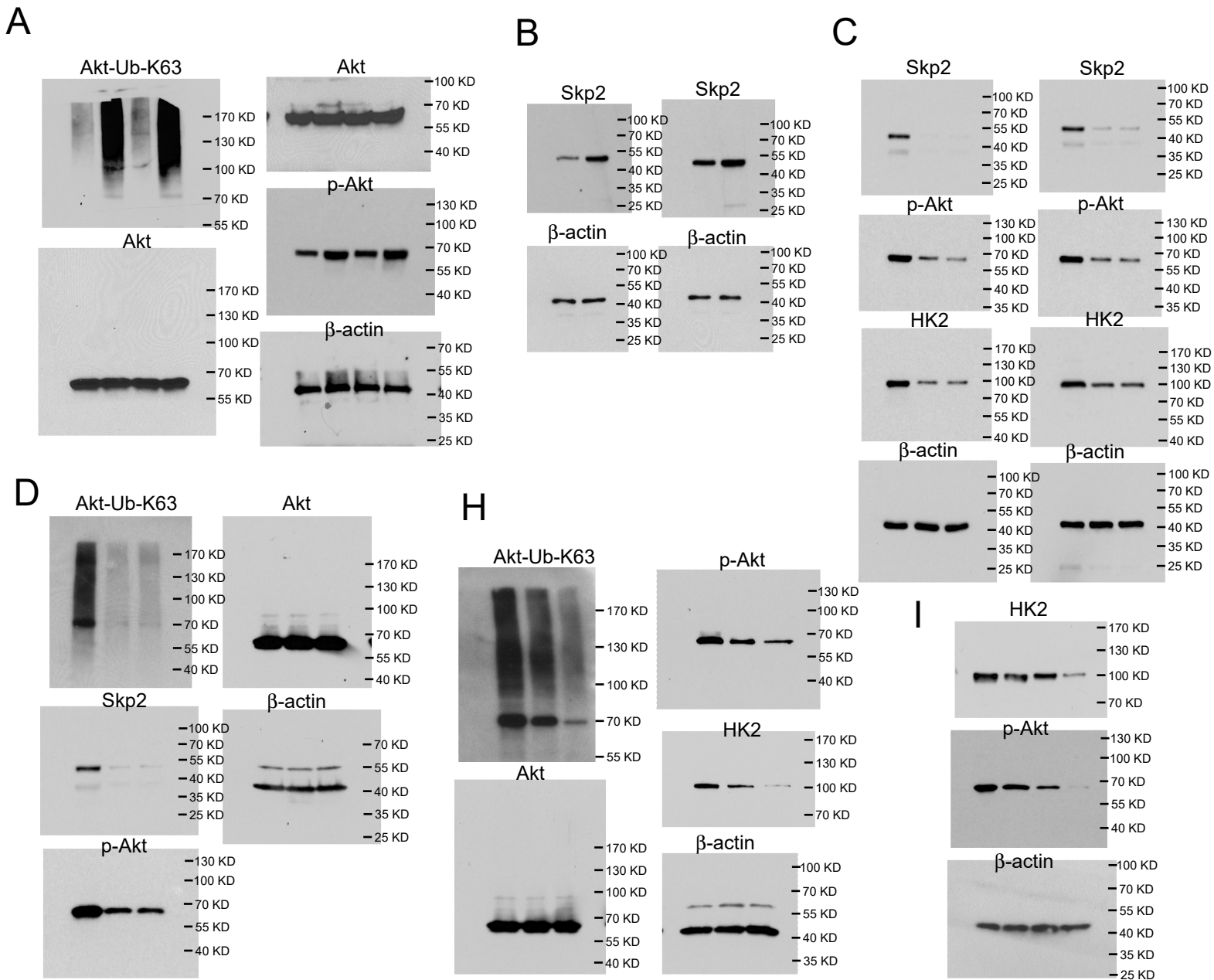

Figure S1

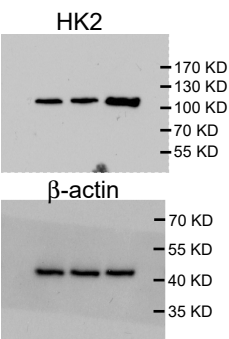

Supplement: Supplementary file 2 — Original Data File [file 41420_2023_1648_MOESM2_ESM.pdf]
